# Effect of Low Doses of Dexamethasone on Experimental Pulmonary Tuberculosis

**DOI:** 10.3390/microorganisms11061554

**Published:** 2023-06-10

**Authors:** Jacqueline V. Lara-Espinosa, María Fernanda Arce-Aceves, Jorge Barrios-Payán, Dulce Mata-Espinosa, Vasti Lozano-Ordaz, Enrique Becerril-Villanueva, María Dolores Ponce-Regalado, Rogelio Hernández-Pando

**Affiliations:** 1 Sección de Patología Experimental, Instituto Nacional de Ciencias Médicas y Nutrición Salvador Zubirán, Vasco de Quiroga 15, Belisario Domínguez Sección 16, Tlalpan, Mexico City 14080, Mexico; jvle_29031991@comunidad.unam.mx (J.V.L.-E.); jorge.barriosp@incmnsz.mx (J.B.-P.); dulmat@yahoo.com.mx (D.M.-E.); lozanoordazvasti@gmail.com (V.L.-O.); 2 Laboratorio de Estudios en Tripasomiasis y Leishmaniasis, Departamento de Inmunología, Instituto de Investigaciones Biomédicas, Universidad Nacional Autónoma de México, Mexico City 04510, Mexico; mariferarce@ciencias.unam.mx; 3 Laboratorio de Psicoinmunología, Instituto Nacional de Psiquiatría Ramon de la Fuente Muñiz, Calzada México-Xochimilco 101, Colonia, Huipulco, Tlalpan, Mexico City 14370, Mexico; 4 Departamento de Ciencias de la Salud, Centro Universitario de los Altos, Universidad de Guadalajara, Av Rafael Casillas Aceves 120, Tepatitlán de Morelos 47620, Mexico; dorisponce61@hotmail.com

**Keywords:** tuberculosis, glucocorticoids, dexamethasone, lung, apoptosis

## Abstract

Tuberculosis (TB) is the deadliest disease caused by a bacterial agent. Glucocorticoids (GCs) have a typical anti-inflammatory effect, but recently it has been shown that they can present proinflammatory activity, mainly by increasing molecules from innate immunity. In the current study, we evaluated the effect of low doses of dexamethasone on *Mycobacterium tuberculosis* in vivo and in vitro. We used an established mice model of progressing tuberculosis (TB) in the in vivo studies. Intratracheal or intranasal dexamethasone therapy administered with conventional antibiotics in the late stage of the disease decreased the lung bacilli load and lung pneumonia, and increased the survival of the animals. Finally, the treatment decreased the inflammatory response in the SNC and, therefore, sickness behavior and neurological abnormalities in the infected animals. In the in vitro experiments, we used a cell line of murine alveolar macrophages infected with *Mtb*. Low-dose dexamethasone treatment increased the clearance capacity of *Mtb* by MHS macrophages, MIP-1α, and TLR2 expression, decreased proinflammatory and anti-inflammatory cytokines, and induced apoptosis, a molecular process that contributes to the control of the mycobacteria. In conclusion, the administration of low doses of dexamethasone represents a promising adjuvant treatment for pulmonary TB.

## 1. Introduction

Tuberculosis (TB) is an infectious disease caused by the bacillus *Mycobacteria tuberculosis* (*Mtb*) [1]. It is considered the oldest pandemic and is the primary cause of death by a single bacterial agent [2]. According to the World Health Organization (WHO), until the SARS-CoV-2 pandemic, TB was the primary cause of death attributed to a single infectious agent, rating above HIV/AIDS. In 2021 there were 1.6 million deaths due to TB and 10.6 million people fell ill with TB [3]. Global TB control is complicated because, in poor countries, which are the most affected by TB, a low-sensitivity microscopy-based 120-year-old diagnosis method and a 100-year-old vaccination are still relied on (Bacille Calmette–Guérin; BCG) [4]. Treatment of active TB currently requires six months of a combination of antibiotics, isoniazid, and rifampicin for six months and pyrazinamide and ethambutol for the first two months of the six-month treatment period [5]. The lengthy course of treatment is a significant barrier to the eradication of TB worldwide. Because of this, the creation of novel medications that can reduce the length of TB treatment is acknowledged as a primary goal of TB drug discovery [5].

The body’s homeostasis is regulated by corticosteroids, which are either synthetic or natural hormones that aid in the body’s resistance to invasion by external agents and changes in the environment. Corticosteroids are classified into glucocorticoids (GCs) and mineralocorticoids [6]. The glucocorticoid receptor (GR), a nuclear receptor member of the family of steroid/thyroid hormone receptors, is where GCs work by binding in the cytosol. In the nucleus, the ligand-bound GR can dimerize and directly bind DNA at recognition sequences known as glucocorticoid response elements (GRE), raising transcription rates [7]. Negative GRE are a unique set of recognition sequences that monomeric GR can bind at DNA, reducing transcription rates. Interfering with the activity of other transcription factors and signaling molecules, most notably NFκ-B, is a major aspect of the mechanism of action of GCs [7]. GCs are widely known and used as antagonistic regulators of inflammation induced by cytokines, and the conventional view suggests that they would antagonize most proinflammatory molecules throughout the genome [8]. However, to improve clinical effectiveness, using high-dose GC therapy results in contraindications such as decreased hypothalamus-pituitary-adrenal axis (HPA) function, weight gain, osteoporosis, and raised blood glucose causing diabetes [9]. Similar to its detrimental effects, GCs have an extensive record of immune system benefits. Studies indicate that GCs can have proinflammatory effects as well [10]. Acute exposure to GCs enhances the peripheral immune response, however, persistent exposure appears to be immunosuppressive. Several Toll-like receptor (TLR) family members, including TLR-2 and TLR-4, are among the genes associated with innate immunity that are expressed because of GC exposure. To strengthen the defense systems and ensure the removal of infections, GCs can work synergistically with proinflammatory mediators such as tumor necrosis factor (TNF) [8,10].

Synthetic GCs, such as dexamethasone (DEX), represent TB’s only clinically approved adjunctive chemotherapeutics [11,12]. GCs are host-directed drugs with a demonstrated beneficial effect on the survival of TB patients [13,14,15,16]. However, the effect of GCs on TB patients remains controversial because there are studies that did not find any beneficial effect of the use of GCs in pulmonary TB [12,17]. Although GCs have been used as adjunctive therapy in TB, the effects of their immunomodulatory activity during mycobacterial infections remain unknown.

Formulations for GCs are available orally, intravenously, intramuscularly, intra-articularly, as aerosols for inhalation, and topically [18]. However, a substantial correlation between the use of oral, inhaled, or systemic GCs and an increase in active TB cases was seen. This link may be due to GCs’ immunosuppressive effects. [17]. It has been acknowledged that treating several diseases with the intranasal (IN) route of administration is more effective. The nasal pathway represents a noninvasive administration route of active pharmaceutical ingredients for local, systemic, and central nervous system (CNS) action and allows the use of fewer doses than other routes of administration [19,20].

Recently, it was demonstrated that low doses of IN administration of DEX in a murine model of pulmonary TB decreased the lung bacterial load, indicating a possible adjuvant effect of DEX in treating TB [21]. In the present work, we evaluate the effect of low doses of DEX in combination with antibiotics (AB) on *Mtb* during late TB using a well-characterized murine model of pulmonary TB [22]. We were also interested in figuring out the possible impact of this alternative therapy on behavioral impairments. Finally, using a cell line of murine alveolar macrophages (Mϕs) infected with *Mtb*, we tried to elucidate the mechanism of action of DEX in the killing of mycobacteria.

## 2. Materials and Methods

### 2.1. Reagents and Materials

OADC (oleic acid, albumin, dextrose, and catalase), as well as the Middlebrook 7H9 and 7H10 media, were purchased from Becton-Dickinson (Detroit, MI, USA). From the American Type Culture Collection (ATCC), the MH-S cell line and *Mtb* reference strain H37Rv were obtained (Manassas, VA, USA). The RPMI-1640 Medium, the resazurin (7-Hydroxy 3H-phenoxazin-3-one-10-oxide sodium salt), and DEX were obtained from Sigma Aldrich (Zwijndrecht, The Netherlands). The fetal bovine serum (FBS) and The Mouse Cytokine Magnetic 20-Plex Panel kit were obtained from Thermo Fisher Scientific (Waltham, MA, USA). The AllPrep^®^ DNA/RNA/Protein Mini Kit, the Omniscript^®^ Reverse Transcription Kit, the Rneasy^®^ Mini Kit for RNA extraction, and the QuantiTectTM SYBR^®^ for RT-PCR were all purchased from Qiagen (Germantown, MD, USA). Primers for the genes analyzed were purchased from InvitrogenTM Thermo Fisher Scientific (Waltham, MA, USA). The remaining reagents and supplies were of analytical grade and were acquired from reliable commercial sources.

### 2.2. Experimental Design

**Figure 1:**
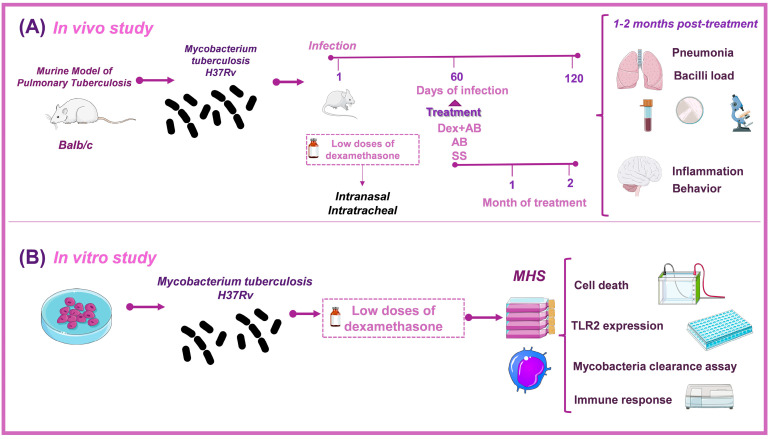
Methodological strategy to analyze the effect of low doses of DEX on *M. tuberculosis*. (**A**) An established murine model of lung TB was used for the in vivo study [22]. Balb/c male mice were 2 months old and were infected with 2.5 × 10^5^ CFU of *Mtb* H37Rv. After 60 days (2 months) of infection, which corresponds to the late stage of the disease, the animals were treated with saline solution, the traditional antibiotics to drug sensible mycobacteria, or DEX plus antibiotics. After 1 and 2 months of treatment, pneumonia, lung bacilli load, survival, behavioral response, and brain inflammation were analyzed by the related methodologies described in the text. (**B**) For the in vitro study, we used a cellular line of alveolar murine Mϕs (MHS), and different concentrations of DEX were proved after the infection with the virulent *Mtb* H37Rv. The effect on cell death, TLR-2 expression, the capacity of mycobacteria clearance by the Mϕs, and the immune response were analyzed by different methodologies described in the text. Parts of the figure were drawn using pictures from Servier Medical Art. Servier Medical Art by Servier is licensed under a Creative Commons Attribution 3.0 Unported License (https://creativecommons.org/licenses/by/3.0/ accessed on 15 January 2023).

We examined the impact of administering low doses of DEX on *Mtb* growth in vitro and in vivo. We used a mouse model of progressive pulmonary TB in the in vivo studies [22]. Animals were infected with *Mtb* H37Rv, starting the treatment with DEX in combination with first-line antibiotics on day 60 postinfection, and euthanized after one and two months post-treatment. Colony-forming units (CFU) were measured in the lungs and right hemispheres of the brain. The lungs were used to evaluate tissue damage (pneumonia) extension. At the same time, we assessed the impact of DEX combined with antibiotics on behavioral anomalies, sickness behavior, and immunological response in various brain regions. The hypothalamus, hippocampus, and frontal cortex were immediately dissected by sectioning with a razor blade following the Mouse Brain in Stereotaxic Coordinates [23]. To evaluate the expression of cytokine genes using RT-PCR, the material was instantly frozen by submersion in liquid nitrogen. Numerous behavioral experiments were conducted. The tests included locomotor activity (LMA) and sickness behavior (body weight loss), anxiety-like behavior, a neurological severity score, depression-like behavior, and short- and long-term memory (Figure 1A). Animals were observed every day, and if any signs of respiratory insufficiency, aggravated cachexia, or complete immobility were seen, they were humanely euthanized under pentobarbital anesthesia. In the in vitro experiments, we investigated the effect of DEX on the cell survival of murine Mϕs, as well as their effect on clearance capability, TLR-2 expression, and cytokine production in noninfected Mϕs and Mϕs infected with H37Rv (Figure 1B). All experiments carried out with infected material were conducted in a P-3 biosecurity level laboratory (BSL-3).

### 2.3. Animals

A total of 72 adult male BALB/c mice were acquired from the INCMNSZ animal house facility, Mexico, when they were eight weeks old. The mice were group-housed (*n* = 5/cage). All the animals were housed in a licensed animal holding facility that ran on a 12:12 h light-dark cycle and was kept at a controlled temperature (23 ± 1 °C) and humidity (50 ± 20%) There was unlimited access to food and drink. All animal studies were carried out under the ARRIVE and Mexican Constitution statute NOM 062-Z00-1999, and were approved by the Internal Committee for the Care and Use of Laboratory Animals of the INCMNSZ in Mexico, protocol number: PAT-1865-16/19-1 (accepted on 7 August 2016).

### 2.4. Murine Model of Pulmonary Tuberculosis

A previous article has covered the mouse model of progressive pulmonary TB [22]. In 7H9 medium with OADC enrichment, the reference *Mtb* strain H37Rv was cultivated. Mid-log-phase cultures were used for all experiments. *Mtb* was counted and stored at −80 °C before use. Frozen bacterial aliquots were thawed and pulse sonicated to remove clumping. After mice infection, the leftover bacterial inoculum was plated to validate the number of CFU administered to the animals. At the age of 8 weeks, male BALB/c mice were anesthetized in a gas chamber with 0.1 mL of sevoflurane per mouse. The trachea was reached by inserting a blunt stainless steel cannula with a small ball at its end through the mouth. The correct intratracheal positioning of the cannula was confirmed by palpating the ball of the cannula against the tracheal rings. The mice were infected with 2.5 × 10^5^ live bacilli via intratracheal instillation, and they were then kept vertical until spontaneous recovery. In an ABSL-3 facility, a total of 72 infected mice were kept in groups of five in cages equipped with microisolators.

### 2.5. Treatments

After 60 days of infection, groups of three mice were treated with 0.05 mg/kg or 0.25 mg/kg of DEX administered through the intranasal route (IN) (20 μL) or intratracheal route (IT) (50 μL) three days per week (Monday, Wednesday, and Friday). The first-line antibiotics rifampicin (RIF), isoniazid (INH), and pyrazinamide were administered to the animals simultaneously at doses of 10 mg/kg, 10 mg/kg, and 30 mg/kg, respectively, in 100 μL of saline solution (SS). As a control, there was a group of animals treated daily with the same first-line antibiotics scheme and a group of infected animals that only received SS. Groups of six mice were exsanguinated under anesthesia with intraperitoneal (i.p.) pentobarbital after 1 and 2 months post-treatment. The right lung and the right hemisphere of the brain were promptly removed, frozen in liquid nitrogen, and utilized for CFU analysis. The left lung was perfused for histopathological examination. Two separate experiments were conducted.

### 2.6. Colony-Forming Units (CFU) Methodology for Determining Brain and Pulmonary Bacillary Loads

In two different trials, the right lungs and hemisphere of the brain from six mice were utilized to count the number of bacterial colonies. In sterile tubes containing 1 mL of isotonic saline solution, brains and lungs were homogenized using a FastPrep homogenizer (MP *Biomedicals*). Homogenates were set in 7H9 medium and serial dilutions were made. Middlebrook 7H10 agar plates were used for plating. CFU was counted after the incubation period of 21 days. The results are presented as the log of CFU per mL.

### 2.7. Preparation of Tissue for Histology and Morphometry

For the histological/morphometric research, the left lungs of six mice per group were perfused (IT) with 100% ethanol. Dehydrated parasagittal sections were divided at a width of 3 m, fixed in paraffin, and stained with hematoxylin and eosin (Oxford Labware, St. Louis, MO, USA). The lungs were recreated using an automated image analyzer system, and the total lung surface area impacted by pneumonia was calculated (Q Win Leica, Milton Keynes, UK). In two independent experiments, six individual mice were used. The data are provided as mean values and SME.

### 2.8. RT-PCR Analysis of TNF and IL12 Expression in Several Brain Areas

The Rneasy Mini Kit (Qiagen, Hilden, Germany) was used to isolate mRNA from the hippocampus, hypothalamus, and frontal cortex of six CT and infected animals at each time point under the instructions provided by the manufacturer. Spectrophotometry (260/280) and agarose gel analysis were used to assess the quantity and quality of RNA. Using 100 μg of RNA, oligo dT, and the Omniscript kit, reverse transcription of the mRNA was carried out. The Quantitec SYBR Green Mastermix kit (Qiagen, Hilden, Germany) and 7500 RT-PCR equipment (Applied Biosystems, Waltham, MA, USA) were used to conduct real-time PCR. Each PCR cycle included negative controls. Primer Express was used to create specific primers for the housekeeping gene glyceraldehyde-3-phosphate dehydrogenase (GAPDH), the TNF, and Interleukin (IL) 12 genes (Applied Biosystems, USA) (Table 1). Initial denaturation was performed at 95 °C for 15 min; then, 40 cycles of 95 °C for 20 s, 60 °C for 20 s, and 72 °C for 34 s were performed. A duplicate of each sample was examined. The 2^−(ΔΔCt)^ technique was used to calculate the fold change in gene expression [24].

### 2.9. Behavioral Studies

The Mice model of progressive TB behavior test methodology was previously published [21,25]. At 24 h before each test the animals were habituated to the test environment. To prevent possible habituation, mice were only evaluated once at the specified time points following treatment. The first four hours of the dark phase of the light cycle were used for all the behavioral tests. A blind observer evaluated and recorded the behavior.

#### 2.9.1. Sickness Behavior

Following 1 and 2 months of treatment, we examined LMA and weight loss to determine sickness behavior. By measuring the mice’s movement over the course of 10 min in an open field, the impact of *Mtb* lung infection on LMA was assessed. The percentage of movement during the 10 min was used to illustrate data. After receiving treatment for 1 and 2 months, an estimation of the weight loss in the *Mtb*-infected animals was made. The animals were weighed each time, and their reduced weight was recorded. In terms of body weight, data are expressed as a percent of body change.

#### 2.9.2. Depression-like Behavior

Using the tail suspension test, we assessed depression-like behavior [26]. The animals were suspended from the tail for 6 min while being monitored for activity, with a particular emphasis on the amount of time the mice were immobile (state of behavioral despair). The duration of the animal’s behavioral desperation during those six minutes was noted.

#### 2.9.3. Anxiety-like Behavior

The I-maze, a modified elevated plus-maze anxiety model in mice, was used to assess anxiety-like behavior [27]. The I-maze’s design consists of a straight wooden passageway that resembles the letter “I”, divided proportionately into three sections: two enclosed areas (closed arms) at either end and an open space in the middle of the two enclosed areas. Animals were under observation for 5 min, and the number of unprotected head dips (uHDIPS), protected head dips (pHDIPS), stretch attend postures (SAP), and time spent in the open areas (%TO) were quantified.

#### 2.9.4. Neurological Outcomes in Animals

A neurological severity score (NSS) was used to assess the motor function and reflexes of the infected mice treated with DEX [28]. The animals were habituated to the boxes where the tests were carried out for 30 min in the morning and in the afternoon, 24 h before the test was carried out. This test consisted of 3 recordings. During the first filming, the animal was placed in a 45 cm × 20 cm × 20 cm plastic box, and its behavior was recorded for 30 s. In the second film, the camera was brought closer to the sides, the head, and the tail, making sure that the movements of the camera were slow, paying more attention to the eyes. In the third recording, the animal was covered with the hand without preventing its escape, and then the sides of the abdominal area (lower abdomen and pelvis) were pressed with the thumb and index finger. A firm abdomen was considered normal, while a flaccid abdomen was considered abnormal. Then, the animal was raised by the tail and slowly lowered until it touched the mat, making sure that its nose was pointing at the camera. Then, it was placed on a grate and the tail was gently pulled, and then it tried to turn on its back on two occasions. The mouse was then placed on a wooden stick at 45° and in a horizontal position 15–20 cm from the ground. The tail was moderately pinched 1 cm from the base for 2 s. Finally, the vibrissae of the animal were touched with a swab, the left flank was pushed, and the swab was moved sideways in front of the head of the mouse without touching it, at a distance between 1 and 2 cm, and the swab was sprayed with 70% alcohol, moving closer to the mouse’s nose. Except for hypomobility, motor impairment, and balance, which were assessed as weak (1), moderate (2), or strong (3), they were valued as absent (0) or present (1). The highest score possible was 31, which denotes neurological impairment. The typical rate ranges from 3 to 6. The complete description of this experiment is shown in Table S1.

#### 2.9.5. Memory Impairment

The short-term and long-term memory were assessed with the object recognition test [29]. In the habituation phase, the animal was left alone in an open field for 10 min so it could become accustomed to the surroundings. At 24 h, the animal was left in the box for 3 min while two identical objects (object A) were positioned in various ways. The short-term memory of the animal was then measured 30 min later. To do this, we placed item A, a known object, in one position and object B, a new object, in the other. During the three minutes that followed, interactions with both objects (the animal sniffing or touching the object with its front legs) were recorded. After 24 h, the long-term memory was assessed. For object B, a fresh object (C) was substituted, and the measurement process was then repeated. The results were displayed using the discrimination ratio, which is the percentage of novel object interactions among all interactions, as specified in the following equation [29].

(1) $$Discrimination\;ratio\;=\frac{novel\;object\;interation}{total\;interations\;with\;both\;objects}$$

### 2.10. Cell and Mycobacteria Culture

The MH-S cell line was expanded and preserved alive in RPMI 1640 with 10% FBS supplement and cultured at 37 °C in 5% CO_2_ conditions. Middlebrook 7H9 supplemented with OADC and 0.5% glycerol was used to cultivate the *Mtb* reference strain H37Rv. It was gently agitated (80 rpm) while growing until it reached the logarithmic growth phase (OD600 = 0.5–0.8).

### 2.11. Infection of Macrophages and Bacillary Load Assay (CFU)

MH-S cells were seeded into 96-well culture dishes at a density of 2 × 10^4^ cells per well and incubated overnight at 5% CO_2_ and 37 °C. The supernatants were discarded before infection and replaced with an FBS-free RPMI medium containing 1 × 10^5^ suspended Mtb (1:5 MOI), which was then incubated for 1 h at 37 °C while being shaken every 15 min. To get rid of free mycobacteria, cells were washed three times with RPMI-1% streptomycin. RPMI–10% FBS (control) or RPMI–10% FBS + DEX 10 μM and 100 μM were added to each well and incubated at 37 °C for 1, 24, 72, and 144 h. Every 36 h the DEX treatment was added to the Mϕs. At each time, cell culture supernatants were collected, and infected Mϕs cells were lysed in 100 μL of 7H9-0.1% sodium dodecyl sulfate (SDS) for 10 min and immediately mixed with 100 μL of 7H9-20% bovine serum albumin (BSA). The lysate was set in 7H9 medium and serial dilutions were made. Middlebrook 7H10 agar plates were used for plating. CFU was counted after the incubation period of 21 days. The results are presented as the log of CFU per mL.

### 2.12. TLR-2 Expression of Macrophages by RT-PCR

The Rneasy Mini Kit was used to isolate mRNA from Mϕs infected with *Mtb* H37Rv and treated for 72 and 144 h with DEX 10 μM and 100 μM, as well as a control with no treatment, following the manufacturer’s instructions. Spectrophotometry (260/280) and agarose gel analysis were used to assess the quantity and quality of RNA. Using 100 μg of RNA, oligo dT, and the Omniscript kit, the mRNA was reverse transcribed. The Quantitec SYBR Green Mastermix kit and 7500 RT-PCR (Applied Biosystems, USA) were used to conduct real-time PCR. Each PCR cycle also contained negative controls. Primer Express was used to create specific primers for the housekeeping gene GAPDH and the TLR-2 gene (Table 1) (Applied Biosystems, USA). The cycling conditions used were the same as described above. The 2^−(ΔΔCt)^ technique was used to calculate the fold change of gene expression [24].

### 2.13. Fragmentation Analysis of DNA

Using DNA electrophoresis on agarose gels, the genomic DNA fragmentation was evaluated. For this purpose, 5 × 10^5^ MH-S cells were grown in 6-well plates, infected with *Mtb* as previously described, and treated with different concentrations of DEX (10–400 μM) up to 24 h; positive controls (cells treated for 24 h with 20 μM H_2_O_2_ or 20 %DMSO) and negative control (untreated control) were also included. A similar experiment was conducted with non-infected cells. After 24 h of treatment, cells were subjected to DNA extraction. DNA isolates were obtained with the AllPrep^®^ DNA/RNA/Protein Mini Kit following the manufacturer’s instructions. The DNA concentration and purity were measured through spectrophotometry (260/280) using an Epoch 2 microplate reader (Agilent Technologies, Santa Clara, CA, USA). A total of 100 ng of each sample was placed onto a 1.5% TAE agarose gel, and SYBR Green staining allowed for the visualization of DNA laddering under a UV transilluminator. A ChemilmagerTM 4400 was used to capture the images (Alpha Innotech, Kasendorf, Germany). All treatments were measured in quadruplicate wells and repeated three times.

### 2.14. Evaluation of the Time-Dependent Viability of Cells

The method for time-dependent viability analysis and the theoretical framework for interpretation were previously disclosed [30]. Briefly, MH-S cells were seeded into 96-well culture dishes at a density of 2 × 10^4^ cells per well and infected with *Mtb* as we previously described. The cells were treated with different concentrations of DEX (10–400 μM) and incubated for 2 or 24 h. The resazurin assay was used to determine cell viability following incubation for 2 or 24 h. Each time, 100 μL of RPMI with 0.5 mM resazurin was added in place of the supernatant, and the cells were then incubated for 24 h at 37 °C. A total of 90 μL of the supernatant was transferred to a fresh 96-well microplate after the incubation period, and a microplate reader was used to measure the absorbance at two wavelengths (540 and 630 nm) (EPOCH2TC BioTech Instruments, Inc., Winooski, VT, USA). A similar experiment was conducted with noninfected cells. Mean viability data were compared to untreated controls (UTC) and corrected for blank values (values lacking cells). Results are represented as mean percentages of untreated controls. All treatments were tested in quadruplicate wells and repeated three times.

### 2.15. Quantification of Secreted Cytokines

Supernatants of infected Mϕs and treated for 144 h with 10 or 100 μM of DEX were collected and centrifuged at 3500 rpm for 5 min and immediately frozen at −70 °C until use. According to the manufacturer’s recommendations, the secreted cytokines analysis was performed using the Mouse Cytokine Magnetic 20-Plex Panel kit (LMC0006M, Life Tech/Invitrogen, Carlsbad, CA, USA). The measurements were completed using Bio-Plex 200 systems (Bio-Rad, Hercules, CA, USA), at the *Red de Apoyo a la Investigación* (RAi) in the National Institute of Medical Science and Nutrition Salvador Zubiran (INCMNSZ). Cytokine concentrations were calculated automatically by the specialized Bio-Plex Manager software version 6.2 software.

### 2.16. Statistical Evaluation

The data for the in vitro studies are shown by the mean and standard error of the mean (SEM) from four different experiments with three replicas. The data for the in vivo experiments are represented by the mean and SEM from 6 distinct mice across two different experiments. All data were gathered in a random order. The data’s normality was assessed using the Shapiro–Wilk normality test. Unpaired *t*-tests (comparison of each group to its respective control) were used to assess the statistical significance of the assessments. The threshold for statistical significance in each experiment was set at *p* < 0.05. The statistical analysis was completed using GraphPad Prism (v 9.4.1) (GraphPad, San Diego, CA, USA).

## 3. Results

### 3.1. Low Doses of Dexamethasone in Combination with Antibiotics Decreased the Lung Bacilli Load and Tissue Damage (Pneumonia) of Mice Chronically Infected with Mtb H37Rv

**Figure 2:**
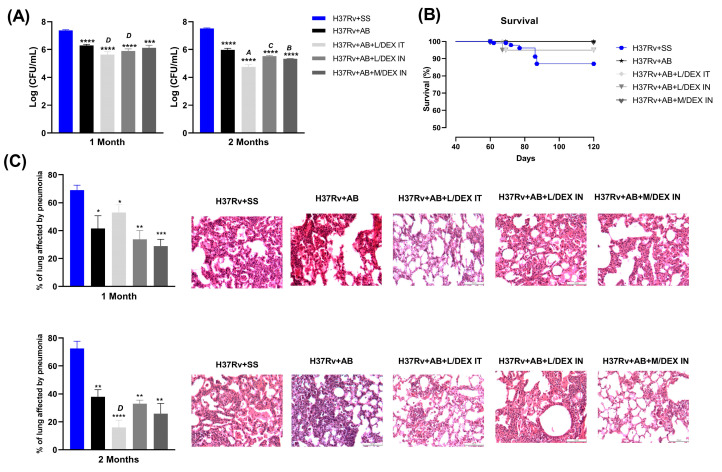
Effect of low (L/DEX) and medium (M/DEX) doses of DEX intratracheal (IT) or intranasal (IN) combined with antibiotics (AB) in the progressive TB model after one or two months of treatment. (**A**) Lung bacilli load. (**B**) Survival rates of the animals. (**C**) Percentage of the lung surface that is impacted by pneumonia and a representative area of the lung 20×. Data are presented as the mean +/− SEM and represent two different experiments (*n* = 6). Unpaired *t*-test. **** *p* < 0.0001, *** *p* = 0.0001, ** *p* = 0.001, * *p* = 0.01 vs. SS control. ^A^ *p* < 0.0001, ^B^ *p* = 0.0001, ^C^ *p* = 0.001, ^D^ *p* = 0.01 vs. AB-treated control.

Previously, we have observed that intranasal (IN) administration of low (0.05 mg/Kg of body weight) (L/DEX) and medium (0.25 mg/Kg of body weight) (M/DEX) doses of DEX reduced the bacterial load in the lungs of mice infected with *Mtb* [21]. Considering the long chemotherapy and the impossibility of treating TB without antibiotics, one interesting possibility is to use agents with immunoregulatory activities, such as DEX, as a coadjuvant to shorten conventional chemotherapy. In the present work, we evaluated the therapeutic effect of IN and intratracheal (IT) administration of DEX in combination with conventional antibiotics (AB) in the late stage of the disease. The results showed that the L/DEX IT and IN plus AB significantly decreased the lung bacterial load compared to the group which only received AB after one and two months of treatment. The treatment with M/DEX IN decreased the lung bacterial load to a greater extent than the AB groups after two months of treatment (Figure 2A). We also evaluated the survival rates of the animals. There were no differences in survival between the group that only received AB and the groups that received DEX (Figure 2B). We additionally evaluated the therapeutic effect of IN and IT treatment of DEX in combination with conventional AB in pneumonia. The results showed that L/DEX and M/DEX IN decreased pneumonia from 41.58% to 33.67% and 28.80%, respectively, in contrast to the group that only received AB after one month of treatment. The L/DEX IT plus AB decreased the lung area affected by pneumonia from 37.84% to 15.90% and the treatment with M/DEX IN plus AB decreased from 37.84% to 25.85% in contrast to the group that only received AB after two months of treatment (Figure 2C). Furthermore, the difference between the DEX groups and the SS control was greater than that between the SS control and AB control. These results showed that DEX has a beneficial effect on the decrease of lung disease. Figure S1 shows the representative micrographs of whole lungs after one and two months of DEX administration.

### 3.2. Low Doses of Dexamethasone in Combination with Antibiotics Decreased the Sickness Behavior of Mice Chronically Infected with Mtb H37Rv

**Figure 3:**
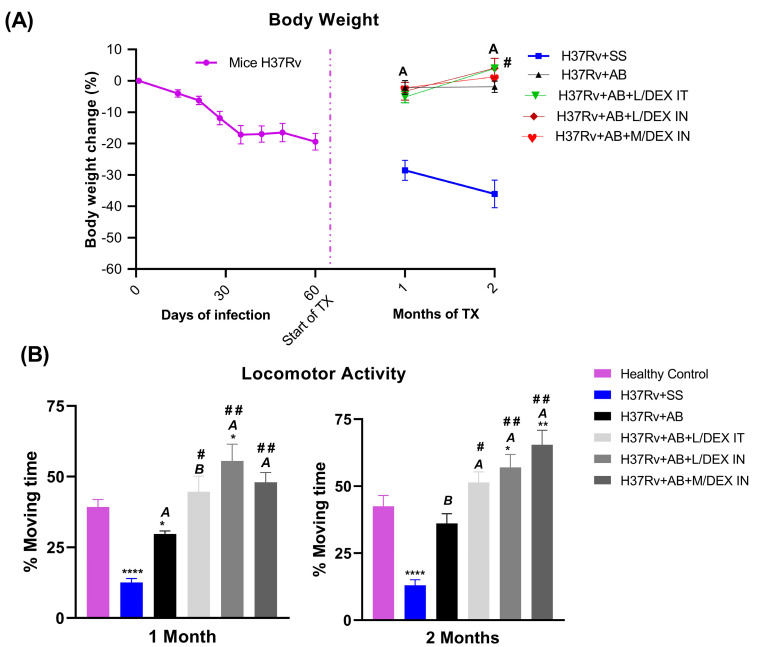
Effect of L/DEX and M/DEX doses combined with AB in sickness behavior in the progressive TB model after one or two months of treatment. (**A**) The animals’ body weight. (**B**) The locomotor activity. Data are presented as the mean +/− SEM and represent two different experiments (*n* = 6). Unpaired *t*-test. **** *p* < 0.0001, ** *p* = 0.001, * *p* = 0.01 vs. healthy control. ^A^ *p* < 0.0001, ^B^ *p* = 0.0001 vs. SS control. ^##^ *p* = 0.001, ^#^ *p* = 0.01 vs. AB control.

Previously, we observed that pulmonary infections with *Mtb* induced sickness behavior and that IN treatment with DEX decreased it [21,25]. Therefore, the effect of DEX with AB on the sickness behavior of mice infected with *Mtb* was evaluated. For this, we assessed the change in body weight and locomotor activity after one and two months of treatment. After 60 days of infection, the animals lost approximately 20% of their body weight when DEX and AB treatment was started. After one and two months of treatment, all the groups increased their body weight in contrast to the SS control. However, there were no differences in body weight between the DEX and AB groups. Only the group that received L/DEX IT plus AB slightly increased the body weight of the infected mice compared to the AB group after two months of treatment (Figure 3A). The locomotor activity was improved from one month of treatment to two months of treatment in the groups that received DEX in combination with AB (Figure 3B). Treatment with DEX plus AB has a stronger effect in reducing sickness behavior than the use of AB alone.

### 3.3. Low Doses of Dexamethasone in Combination with Antibiotics Decreased the Anxiety-like Behavior, Depression-like Behavior, and Neurological Damage, and Improved Memory of Mice Chronically Infected with Mtb H37Rv

**Figure 4:**
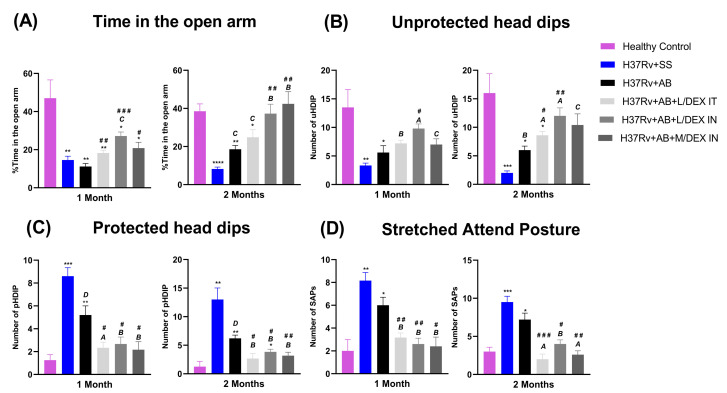
Effect of L/DEX and M/DEX doses combined with AB in anxiety-like behavior in the progressive TB model evaluated in the elevated I-maze after one or two months of treatment. (**A**) Time spent in the open arm. (**B**) Unprotected head dips. (**C**) Protected head dips. (**D**) Stretched attend posture (SAP). Data are presented as the mean +/− SEM and represent two different experiments (*n* = 6). Unpaired *t*-test. **** *p* < 0.0001, *** *p* = 0.0001, ** *p* = 0.001, * *p* = 0.01 vs. healthy control. ^A^ *p* < 0.0001, ^B^ *p* = 0.0001, ^C^ *p* = 0.001, ^D^ *p* = 0.01 vs. SS control. ^###^ *p* = 0.0001, ^##^ *p* = 0.001, ^#^ *p* = 0.01 vs. AB control.

As we have previously demonstrated that *Mtb* induced anxiety-like behavior and DEX administrated IN alone decreased it [21,25], we wanted to evaluate the effect of DEX with AB in chronically infected mice. The anxiety-like behavior was evaluated after one and two months of treatment with an elevated I-maze [27]. The results showed that the animals treated with DEX IN and IT spent more time in the open arm of the maze and had more unprotected head dips than the animals which only received AB, both parameters that indicate an anxiolytic effect. On the other hand, the treatment decreased protected head dips and stretched attend posture, parameters that indicate anxiogenic activity (Figure 4). In general, we can say that treatment with DEX plus AB has a stronger effect in reducing anxiety-like behavior than the administration of AB alone.

**Figure 5:**
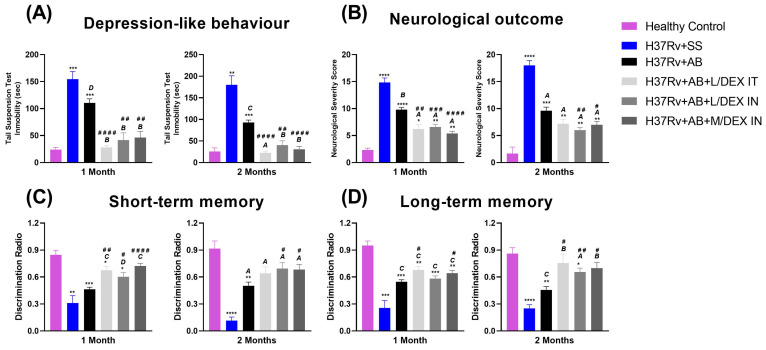
Effect of L/DEX and M/DEX doses combined with AB in depression-like behavior, neurological damage, and memory in the progressive TB model after one or two months of treatment. (**A**) Depression-like behavior. (**B**) Neurological severity score. (**C**) Short-term memory. (**D**) Long-term memory. Data are presented as the mean +/− SEM and represent two different experiments (*n* = 6). Unpaired *t*-test. **** *p* < 0.0001, *** *p* = 0.0001, ** *p* = 0.001, * *p* = 0.01 vs. healthy control. ^A^ *p* < 0.0001, ^B^ *p* = 0.0001, ^C^ *p* = 0.001, ^D^ *p* = 0.01 vs. SS control. ^####^ *p* < 0.0001, ^###^ *p* = 0.0001, ^##^ *p* = 0.001, ^#^ *p* = 0.01 vs. AB control.

Previously, we have observed that lung infection with *Mtb* increased the presence of depression-like behavior and decreased short- and long-term memory [25]. The IN administration of DEX alone decreased these behavioral abnormalities [21]. Therefore, in the present work, we evaluated the effect of the treatment with DEX in combination with AB in the late stage of pulmonary disease. The results showed that DEX plus AB had a stronger antidepressive effect than AB alone after one and two months of treatment. The L/DEX IT treatment was the most effective for decreasing depression-like behavior, but the IN route was also effective (Figure 5A). The treatment with DEX decreased the neurological severity score (NSS) of the infected animals to a greater extent than the group which only received AB (Figure 5B). Similar results were found in short- and long-term memory. The L/DEX IT decreased damage in the long-term memory to a greater extent than the L/DEX and M/DEX IN (Figure 5C,D). Taken together, these results demonstrate that L/DEX IT plus AB has a beneficial effect on the behavioral abnormalities present in the mice infected with *Mtb*.

### 3.4. Low Doses of Dexamethasone in Combination with Antibiotics Decreased the Expression of IL-12 and TNF in Different Brain Areas of Mice Chronically Infected with Mtb H37Rv

**Figure 6:**
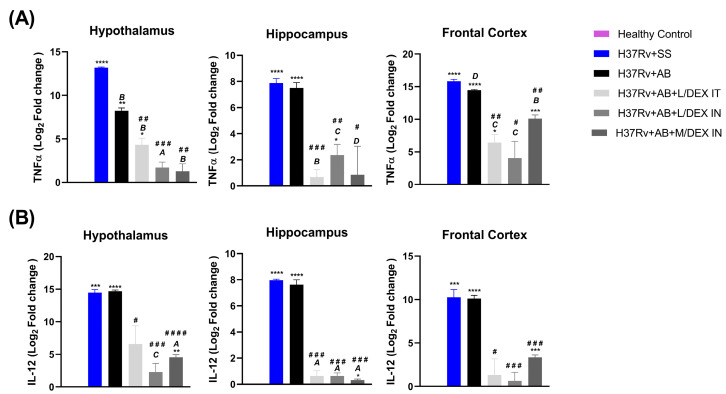
Effect of L/DEX and M/DEX doses combined with AB in the expression of TNF and IL-12 of the hypothalamus, hippocampus, and frontal cortex in the progressive TB model after two months of treatment. (**A**) TNF expression. (**B**) IL-12 expression. Data are presented as the mean +/− SEM and represent two different experiments (*n* = 6). Unpaired *t*-test. **** *p* < 0.0001, *** *p* = 0.0001, ** *p* = 0.001, * *p* = 0.01 vs. healthy control. ^A^ *p* < 0.0001, ^B^ *p* = 0.0001, ^C^ *p* = 0.001, ^D^ *p* = 0.01 vs. SS control. ^####^ *p* < 0.0001, ^###^ *p* = 0.0001, ^##^ *p* = 0.001, ^#^ *p* = 0.01 vs. AB control.

As we have previously observed that *Mtb* induces neuroinflammation and IN DEX treatment decreased it [21,25], in the present work we evaluated the effect of the treatment with DEX in combination with AB in TNF and IL-12 expression in the hypothalamus, hippocampus, and frontal cortex of chronically infected mice. The results showed that TNF decreased in the brain areas studied after DEX IN and IT treatment. This change was more significant in the groups of DEX plus AB than in the group which only received AB (Figure 6A). In the case of IL-12, we observed that the administration of AB alone did not decrease the expression of this cytokine in the hypothalamus, hippocampus, and frontal cortex. In contrast, the administration of AB in combination with IT or IN DEX decreased the expression of IL-12 significantly; the decrease was greater in the hippocampus (Figure 6B).

### 3.5. Low Doses of Dexamethasone Decreased the Bacilli Load and Induced TLR-2 Expression of the Alveolar Macrophages MHS Infected with Mtb H37Rv

**Figure 7:**
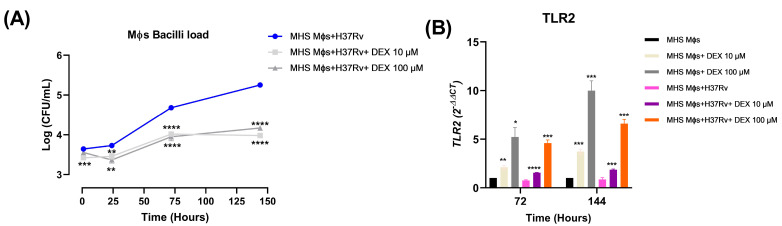
Effect of the treatment with different doses of DEX on the bacilli load and the *TLR-2* expression of MHS Mϕs infected with *Mtb*-H37Rv. After 1 h of infection, the treatment with DEX started and was added every 36 h to the Mϕs. (**A**) Mϕs bacilli load (CFU). (**B**) *TLR-2* expression. Data are represented as mean +/− standard error of the mean (SEM) and represent three different experiments (*n* = 12). Unpaired *t*-test against not treated control * *p* = 0.01, ** *p* = 0.001, *** *p* = 0.0001, **** *p* < 0.0001.

Since, in the first part of this work, we observed that the administration of DEX decreased the bacillary load in a model of pulmonary TB, in the second part of this work we tried to determine the mechanism by which this decrease is being induced. For this, we first tested the direct effect of DEX on mycobacterial growth in a minimum inhibitory concentration assay. DEX did not affect *Mtb* directly. Therefore, we evaluated the effect of two low doses of DEX (10 and 100 μM) in the bacilli load of alveolar Mϕs infected with *Mtb* H37Rv in a kinetic of 1, 24, 72, and 144 h. In the first hour following infection, Mϕs treated with DEX 10 μM had fewer intracellular bacteria than the untreated control group. At 24, 72, and 144 h, both DEX treatments significantly decreased bacillary load compared to the untreated group. The greatest effect was observed at 144 h postinfection (Figure 7A). Both doses significantly decreased the bacterial load of infected Mϕs, contributing to the microbicidal capacity of these cells.

According to reports, GCs could enhance the production of innate immune response components such as TLR-2 [10]. Therefore, we evaluated the effect of DEX on the expression of TLR-2 in alveolar Mϕs not infected and infected with *Mtb* by RT-PCR. Treatment with 10 and 100 μM DEX significantly increased TLR-2 expression in both noninfected and infected Mϕs at 72 and 144 h. The increase in TLR-2 was greater in noninfected Mϕs than in infected Mϕs (Figure 7B).

### 3.6. Dexamethasone Induces Apoptosis in Alveolar Macrophages Not Infected and Infected with Mtb H37Rv

**Figure 8:**
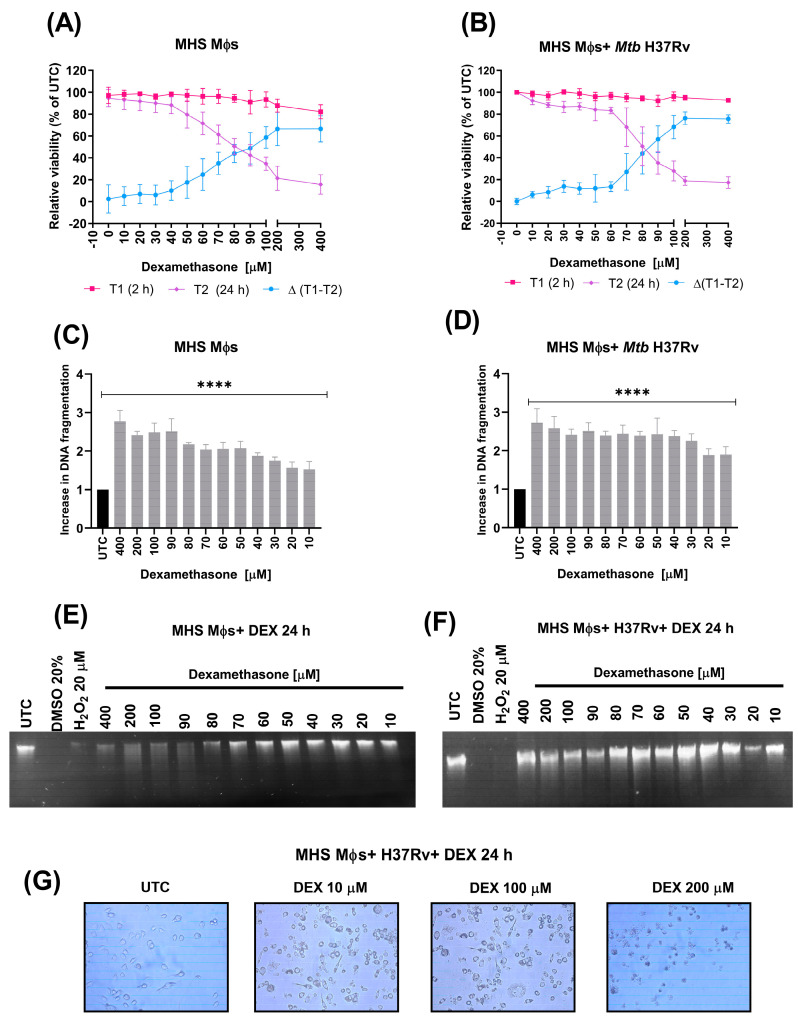
Effect of different doses of DEX on the viability of alveolar murine Mϕs not infected and infected with *Mtb*. (**A**,**B**) Effect of different doses of DEX on the viability of MHS Mϕs analyzed by the resazurin viability-based cell death discrimination analysis. (**C**,**D**) After treatment with different doses of DEX, the apoptotic DNA fragmentation of MHS Mϕs was qualitatively analyzed by agarose gel electrophoresis, quantified by Image J, and normalized to the untreated control (UTC). DMSO 20% and H_2_O_2_ 20 μM were used as necrosis control. (**E**,**F**) Representative image of the agarose gel electrophoresis of DNA fragmentation of MHS Mϕs after treatment with different doses of DEX. (**G**) Microscopy images 20× of MHS cells infected with *Mtb* H37Rv not treated and treated with 10, 100, or 200 µM DEX for 24 h. The data is presented as the mean +/− SEM and represents three independent experiments (*n* = 12). Unpaired *t*-test against nontreated control **** *p* < 0.0001.

We then evaluated the effect of DEX on the viability of *Mtb*-infected and noninfected alveolar Mϕs. For this, we used the resazurin-based cell death screening method [30]. The results showed that in the early reading (2 h) there was no decrease in viability in the infected and noninfected Mϕs, even at the highest DEX concentrations. In contrast, viability measured at 24 h post-treatment decreased for all treatments in the infected and noninfected cells. Proliferation, apoptosis, and necrosis can be distinguished from one another using the calculation of the difference curve of two different reading time points [30]. To determine whether cells were dying, we calculated the difference curve (Delta) between the 2 and 24 h data. The curve indicated positive values starting at the lowest concentrations. At 200 μM, the values reached their peak. These findings point to apoptosis, with strong metabolic activity at early stages and decreased activity at late stages (Figure 8A,B). The induction of apoptosis was also evaluated with the detection of an apoptotic DNA ladder on 1.5% agarose gel. The results showed that DEX induced DNA fragmentation in a dose-dependent manner. This was observed in both the noninfected Mϕs and the infected Mϕs with *Mtb.* However, the infected Mϕs presented lower DNA fragmentation than the noninfected cells, indicating that *Mtb* decreases the apoptosis induced by DEX (Figure 8C–F). Additionally, it was visible under a microscope that apoptosis and cell death had been induced. When cells were seeded, allowed to grow for 24 h, then infected with *Mtb* and treated with 10, 100, or 200 μM DEX for another 24 h, an increasing number of shrunken and dead cells started to show up in the DEX-treated samples (Figure 8G).

### 3.7. Dexamethasone Reduces the Anti-Apoptotic Proteins FGFβ, VEGF, and IL6, Reduces Pro- and Anti-Inflammatory Cytokines, and Increases MIP-1α in Alveolar Macrophages Infected with Mtb H37Rv

As we wanted to determine the molecular changes induced by DEX in the alveolar Mϕs infected with *Mtb*, we quantified the production of proteins related to the immune response in the supernatants of these cells after 144 h of treatment. The infection with *Mtb* increased the Fibroblast growth factor β (FGF-β), Interleukin (IL) 6 (IL-6), and Vascular endothelial growth factor (VEGF). The treatment with DEX reduced these proteins that have been related to mitogenic activity (Table 2).

The production of anti-inflammatory cytokines was also measured. The infection with *Mtb* increased the production of IL-10, IL-13, and IL-5. The treatment with DEX at 10 and 100 μΜ significantly reduced these cytokines. Similar results were observed with proinflammatory cytokines. The infection with *Mtb* increased the production of IL-1β, IL-1α, IL-12, Interferon γ (IFN-γ), TNF, and the Granulocyte macrophage colony stimulating factor (GM-CSF). The treatment with DEX at 10 and 100 μΜ reduced these cytokines notably. Interestingly, the macrophage inflammatory protein-1 alpha (MIP-1α) was decreased by *Mtb* infection and DEX treatment increased the levels of this protein significantly. It seems that DEX treatment avoids the production of not only proinflammatory cytokines but also anti-inflammatory cytokines, turning the Mϕs into unpolarized phenotype (M0) (Table 2).

## 4. Discussion

TB is the principal cause of bacterial infectious disease death worldwide [3]. Moreover, MDR TB is still one of the greatest dangers to containing the TB epidemic [31]. Repurposing medicines that were developed for indications other than TB is an economical strategy to bridge the time until novel drugs become available [32]. Despite GCs’ significance in reducing inflammatory reactions, it is unclear how they affect the host’s immune system and defenses against bacterial pathogen invasion. Additionally, the molecular foundation of GCs’ positive effects in the management of bacterial illnesses such as TB is also unknown. Due to its low price, numerous formulations, well-known mechanism of action, and various administration methods, DEX has gained a large amount of interest. [33]. Here, we evaluated the effect of low doses of DEX on *Mtb* in vivo and in vitro.

We used a murine model of pulmonary TB for the in vivo tests. [22]. Following a therapeutic strategy, the treatments with low doses of IN and IT DEX in combination with AB started after two months of infection, when the disease was in the late stage. The results showed that low doses of DEX in combination with AB decreased the lung bacilli load and pneumonia after two months of treatment to a greater extent than the administration of AB alone in chronically infected mice. In TB, tissue pathology frequently occurs from an overactive inflammatory response, which causes persistent inflammation and tissue dysfunction [34]. According to the results, the IT and IN treatment with DEX decreased the induction of tissue damage. Similar results have been found in a hamster model of infection with SARS-CoV-2, where the treatment with DEX intraperitoneal (1 mg/Kg per dose) decreased severe pneumonia by decreasing proinflammatory cytokines [35]. In another work, using an animal model of rabbits infected with *Mtb* CDC1551, the intramuscular administration of 10 mg/Kg/day of DEX increased bacillary load in the lung; however, the number of visible tubercles was lower [36]. In an investigation of *Staphylococcus aureus*-induced experimental endophthalmitis, antibiotic and DEX therapy reduced inflammation in comparison to DEX or antibiotics alone, preserving retinal function and minimizing tissue damage. The combined therapy also increased bacterial clearance as compared to antibiotics alone [37]. This information suggests that DEX decreases chronic, nonproductive inflammation and improves the host antimicrobial response, as we observed in the animals with TB, but the DEX dose should be low—a high DEX dose (2.5 mg/kg) produced extensive tissue damage, high bacillary loads, and dissemination [21].

TB is characterized by an excessive systemic inflammatory response [38]. Systemic inflammation produces neuroinflammation and neuronal damage caused by oxidative stress and activating microglia that impair cognitive function, including learning and memory, and can contribute to neurobehavioral changes, including the development of neuropsychiatric disorders [39,40,41]. In the absence of brain bacteria, we have shown that *Mtb* lung infection caused neuroinflammation and behavioral impairments [25], and similar results have been described in *Mycobacterium lepraemurium* [42,43]. The results of this work showed that low doses of DEX IT and IN in combination with AB reduced sickness behavior, reduced anxiety-like behavior, decreased depression-like behavior, improved neurological outcomes, and enhanced short- and long-term memory in TB mice. Interestingly, the improvement in behavior was greater in the DEX groups than in the AB alone group. The underlying mechanism was thoroughly related to the inhibition of neuroinflammation. Animals treated with DEX and AB showed a decrease in the expression of TNF and IL-12 in the hippocampus, hypothalamus, and frontal cortex. On the other hand, animals who received only AB presented similar expressions of these proinflammatory cytokines to the SS control. Related results have been found in patients with underlying TB meningitis, who after six months of antibiotic therapy presented elevated levels of TNF and IFN-γ, indicating the persistence of neuroinflammation [44]. This suggests that the severity of mycobacterium infection results predominantly from the immune response rather than the organism itself. In this context, the administration of anti-inflammatory molecules such as DEX in controlled doses with conventional AB therapy is a promising strategy to manage excessive inflammation and the number of bacteria.

We evaluated two different routes of administration of DEX: IT and IN. In terms of behavioral alterations, there were no differences between the routes of administration. However, the mechanism of decreasing neuroinflammation and consequently behavior alterations could be not the same. The IN route is a viable option for drug delivery into the CNS [20]. This route allows the transport of GCs directly to the CNS through the olfactory and trigeminal nerves, improves the drug bioavailability in the brain, and decreases degradation as well as consumption of the drug through systemic clearance [45,46]. There is robust evidence of the effectiveness of the IN administration of GCs in diseases related to neuroinflammation. Some examples included neuroinflammation induced by systemic injection of LPS, a model of stroke induced by occlusion of the middle cerebral artery for 60 min, a model of experimental autoimmune encephalomyelitis (EAE), and a model of chronic exposure to toluene. In all these models, IN administration of GCs decreased neuroinflammation, emphasizing that the IN route is more effective in decreasing neuroinflammation than the intravenous route [19,20,47,48], and in mice and humans, a larger quantity of GCs reaches the brain via IN administration than via intravenous administration [49,50]. The nose is also an external conduit to the lungs and a practical route for the topical and systemic administration of medications due to its anatomical position and physiological characteristics (surface area, innervation, and blood supply) [51]. In a murine model of lung damage induced by silica particles, the IN administration of flunisolide decreased lung inflammation and damage and increased the elimination of the silica particles [52]. In our model of pulmonary TB, the IN administration of DEX decreased neuroinflammation and had a beneficial effect on the lungs, demonstrating that the IN route is optimal to treat inflammation in the CNS of TB mice.

Local IT instillation or aerosol administration is an attractive route of treatment, since the respiratory tree is an enclosed environment, and the lungs are the target organ for treatment [53]. Interestingly, the lung bacterial load and pneumonia decrease induced by the DEX administration in the TB mice was higher in the IT group. This could be due to the direct anti-inflammatory effect of DEX on the infectious site. The effect of decreasing neuroinflammation could be related to the communication between the lungs and the brain via the vagus nerve. The larynx, tracheobronchial tree, and lungs receive most of their sensory neurological signals from the vagus nerve [54]. The body launches an immune response to get rid of invasive germs for lung conditions such as lung infections. However, overactive immune systems could injure the CNS. Circulating inflammatory markers could invade CNS through different mechanisms, including vagus nerve stimulation, activating glial cells, and exacerbating nerve cell death [55]. Therefore, decreasing lung inflammation represents a pathway to decrease neuroinflammation as well.

In the in vitro experiments, we used a cell line of alveolar murine Mϕs infected with *Mtb* to assess the effect of low doses of DEX on *Mtb*. Even though GCs have a systemic and complex impact on human and mouse physiology, the performed in vitro experiments can improve the understanding of the host response to mycobacterial infection on a cellular level. The results showed that 10 or 100 μM DEX decreased the intracellular bacterial load of infected Mϕs. Similar results have been found in human monocyte-derived Mϕs infected with *Mtb* and treated with different corticosteroids, including DEX. All the corticosteroids tested reduced intracellular bacterial growth [56]. This demonstrated that DEX could decrease intracellular *Mtb* growth not only in mice Mϕs but also in human Mϕs, which is promising in the treatment of TB.

The reduction of intracellular *Mtb* in alveolar Mϕs coincided with an increase in the expression of TLR-2. It is well known that TLR-2 may be one of the protective immunoregulatory mechanisms involved in host defense against many bacterial pathogens [57]. In previous work, it has been demonstrated that DEX synergistically enhanced TLR-2 expression in combination with TNF and IFNγ in human respiratory epithelial cells [58]. TLR-2 expression is induced by the transcriptional interaction between NF-κB and GR, which both bind to the TLR-2 promoter, combined with inhibition of p38α MAPK via the specific up-regulation of the MAPK phosphatase-1 (MKP-1) [59]. Moreover, it has been demonstrated that DEX inhibits the necrotic cell death of *Mtb*-infected MRC-5 human lung fibroblasts by facilitating MKP-1-dependent dephosphorylation of p38 MAPK [11]. It seems that the up-regulation of MKP-1 increases TLR-2 expression and avoids necrosis which could be related to the intracellular control of *Mtb* growth that was observed in the present work; however, more experiments and evidence are necessary to corroborate this. Although there was an up-expression of TLR-2 of the *Mtb*-infected Mϕs treated with DEX, the pro- and anti-inflammatory cytokines levels were decreased, which has been described before and indicates that TLR-2 induction did not enhance TLR-2 signaling [60].

Interestingly, DEX significantly increased the levels of MIP-1α in the infected Mϕs with *Mtb*. Similar results have been found in a murine model of acute lung inflammation induced with LPS and ozone exposure, where the administration of DEX significantly induced the expression of chemokines including MIP-1α. Moreover, DEX increased the immune cell presented in the broncho-alveolar lavage of the animals [61]. This showed that DEX not only induced MIP-1α in vitro but in vivo as well. MIP-1α is an inflammatory chemokine produced by cells during infection or damage and is important for the recruitment of Mϕs and T lymphocytes from circulation to sites of infection or injury [62]. Therefore, the increase of MIP-1α could be related to better control of *Mtb*; however, more research is needed to understand how GCs induce the expression of chemokines in the context of infection.

The results also showed that DEX induced apoptosis of the *Mtb*-infected Mϕs and decreased pro- and anti-inflammatory cytokines. Another work showed that DEX induced apoptosis of lipopolysaccharide-stimulated rat alveolar Mϕs and decreased proinflammatory cytokines [63]. The induction of apoptosis of the *Mtb*-infected Mϕs by DEX was related to a reduction in the levels of IL-6, FGF-β, and VEGF, proteins with antiapoptotic effects [64,65,66]. By preventing the release of intracellular pathogens and the propagation of mycobacterial infection, apoptosis plays a crucial part in the host’s defenses against intracellular infections such as *Mtb* [67]. Therefore, the induction of Mϕs apoptosis could be related to the reduction of intracellular mycobacteria.

In summary, the present work demonstrated that low doses of DEX in combination with AB synergized the decrease of bacterial load in the lungs and the area of the lungs affected by pneumonia, in addition to having a beneficial effect on the CNS, and decreasing neuroinflammation and behavioral alterations in vivo. The effect of DEX decreasing the bacillary load was also verified in vitro. This was related to an increase in TLR-2 and MIP-1α and the induction of apoptosis of the infected Mϕs. These data show that low and controlled doses of DEX could be used as an adjuvant to conventional chemotherapy. However, all the results of this work were completed with *Mtb* H37Rv which develops moderate disease in BALB/c mice. The role of this treatment has yet to be explored in a larger number of *Mtb* strains including hypervirulent strains such as the Beijing genotype; future work will provide a broader picture of the use of DEX as an adjuvant treatment in TB.

## 5. Conclusions

**Figure 9:**
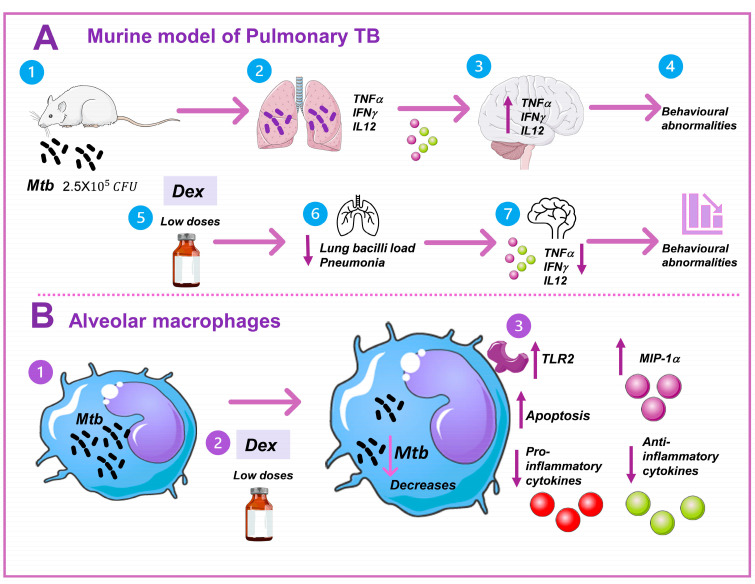
Low doses of DEX decreased *Mtb* load in vitro and in vivo. (**A**) In the murine model of pulmonary TB, intratracheal *Mtb* infection (2) led to the development of active disease in the mice. A strong inflammatory response to mycobacteria in the lungs causes (3) neuroinflammation through humoral and neuronal pathways, which is characterized by an increase in the production of different cytokines. (4) This results in behavioral changes and neuropsychiatric symptoms such as depression and anxiety. (5) The administration of a low dose of DEX in combination with antibiotics decreased (6) the bacilli load and pneumonia to a greater extent than the antibiotics alone. (7) This was related to a decrease in the inflammatory response in the CNS and therefore a decrease in behavioral abnormalities. (**B**) In the in vitro experiments, (1) we infected murine alveolar Mϕs with *Mtb*, (2) and treated it with low doses of DEX. (3) The treatment with DEX in alveolar Mϕs induced the expression of TLR-2, decreased the production of both pro- and anti-inflammatory cytokines, decreased proteins related to cell survival, increased MIP-1α, and induced apoptosis. Together they decreased the bacilli load. In conclusion, the results of the present work showed that DEX could be used as an adjuvant in the treatment of pulmonary tuberculosis. Parts of the figure were drawn using pictures from Servier Medical Art. Servier Medical Art by Servier is licensed under a Creative Commons Attribution 3.0 Unported License (https://creativecommons.org/licenses/by/3.0/ accessed on 15 January 2023).

DEX significantly inhibits bacterial growth in vitro and in vivo at low dosages. In the current study, we demonstrated that administering DEX via the IN or IT route in conjunction with antibiotics decreased the lungs’ bacillary load and the area of pneumonia, and tended to increase the survival of infected animals in a murine model of pulmonary TB. In addition, DEX therapy reduced TNF and IL12 gene expression in the frontal cortex, hypothalamus, and hippocampus of mice with intratracheal *Mtb* infection. The treatment lessened the behavioral alterations in mice with TB, including sickness behavior, depressive and anxious states, neurological impairment, and memory loss. In an in vitro model, low doses of DEX reduced the bacilli load, induced the expression of TLR-2, induced the production of MIP-1α, reduced the production of pro- and anti-inflammatory cytokines of infected alveolar Mϕs, and induced apoptosis. Low dosages of DEX could therefore be utilized as an adjuvant in the treatment of TB (Figure 9).

## Figures and Tables

**Table 1:** Gene expression was assessed using the following primer sequences.

Gene	Forward	Reverse
GAPDH	5′-CATTGTGGAAGGGCTATGA-3′	5′-GGAAGGCCATGCCAGTGAGC-3′
TLR-2	5′-TGCTTTCCTGCTGGAGATTT-3′	5′-TGTAACGCAACAGCTTCAGG-3′
TNF	5′-GCCGAGAAAGGCTGCTTG-3′	5′-TGTGGCTTCGACCTCTACCTC-3′
IL12	5′-GGATGGAAGAGTCCCCCAAA-3′	5′-GCTCTGCGGGCATTTAACAT-3′

**Table 2:** Effect of low doses of DEX on the levels of proteins of alveolar murine Mϕs infected with *Mtb*-H37Rv. Supernatants from Mϕs infected with *Mtb* and treated with 10 or 100 μM for 144 h were used for quantification using the Mouse Cytokine Magnetic 20-Plex Panel kit. The data are represented as mean ± SEM. Unpaired *t*-test. * *p* = 0.01, ** *p* = 0.001, *** *p* = 0.0001, **** *p* < 0.0001 vs. Mϕs. ^D^ *p* = 0.01, ^C^ *p* = 0.001, ^B^ *p* = 0.0001, ^A^ *p* < 0.0001 vs. Mϕs + H37rv.

Protein (pg/mL)	Mϕs	Mϕs + H37Rv	Mϕs + H37Rv + DEX 10 μM	Mϕs + H37Rv + DEX 100 μM
Mitogenic activity				
FGF-β	82.29 ± 3.225	97.99 ± 2.751 **	72.26 ± 7.10 ^C^	64.24 ± 2.232 **^/A^
IL-6	241.6 ± 54.59	628.74 ± 53.37 ***	132.5 ± 18.46 ^A^	101.40 ± 16.04 *^/A^
VEGF	1203 ± 215.5	2761 ± 584.7 *	23.06 ± 6.645 ***^/B^	31.68 ± 12.20 ***^/B^
Anti-inflammatory				
IL-10	31.19 ± 6.051	54.16 ± 3.566 **	35.24 ± 6.283 ^D^	20.10 ± 3.164 ^A^
IL-13	18.00 ± 2.843	26.13 ± 2.45	4.709 ± 0.5931 ***^/A^	5.753 ± 1.085 **^/A^
IL-5	13.96 ± 0.9218	14.53 ± 1.057	8.042 ± 1.809 *^/D^	8.377 ± 0.7788 **^/B^
Proinflammatory				
IL-1β	84.58 ± 5.483	105.2 ± 3.227 **	80.93 ± 5.02 ^C^	71.04 ± 4.384 ^A^
IL1-α	45.25 ± 8.864	114.3 ± 11.05 **	23.64 ± 7.257 ^A^	10.04 ± 3.411 **^/A^
IL-12	17.83 ± 2.464	28.01 ± 3.055 *	11.75 ± 2.72 ^C^	9.51 ± 3.018 ^C^
IFN-γ	9.482 ± 1.926	15.22 ± 1.042 *	5.40 ± 2.435 ^C^	1.837 ± 0.6193 **^/A^
TNF	88.32 ± 11.34	130.5 ± 9.615 *	81.81 ± 11.83 ^C^	60.49 ± 17.24 ^C^
GM-CSF	16.05 ± 2.223	73.44 ± 24.63	26.85 ± 7.354	7.700 ± 1.974 *^/D^
MIP-1α	20.86 ± 2.641	5.685 ± 1.644 ***	16338 ± 25.40 ****^/A^	17726 ± 18.75 ****^/A^

## Data Availability

The data are accessible upon request to the corresponding authors.

## Supplementary Materials

The following supporting information can be downloaded at: https://www.mdpi.com/article/10.3390/microorganisms11061554/s1, Figure S1: Effect of L/DEX and M/DEX doses combined with AB in the lung area affected by pneumonia in the progressive TB model. Representative micrographs of whole lungs after one (A) and two months (B) of DEX administration; Table S1: Neurological Severity Score (NSS) for mice.
