# Supplementary material for: Effect of Low Doses of Dexamethasone on Experimental Pulmonary Tuberculosis
Source: Microorganisms. 2023 Jun 10;11(6):1554. doi: 10.3390/microorganisms11061554 (PMC10305529; doi:10.3390/microorganisms11061554)
Supplement: Supplementary file 1 [file microorganisms-11-01554-s001.zip › microorganisms-2424687-supplementary.pdf]

microorganisms

Supplementary

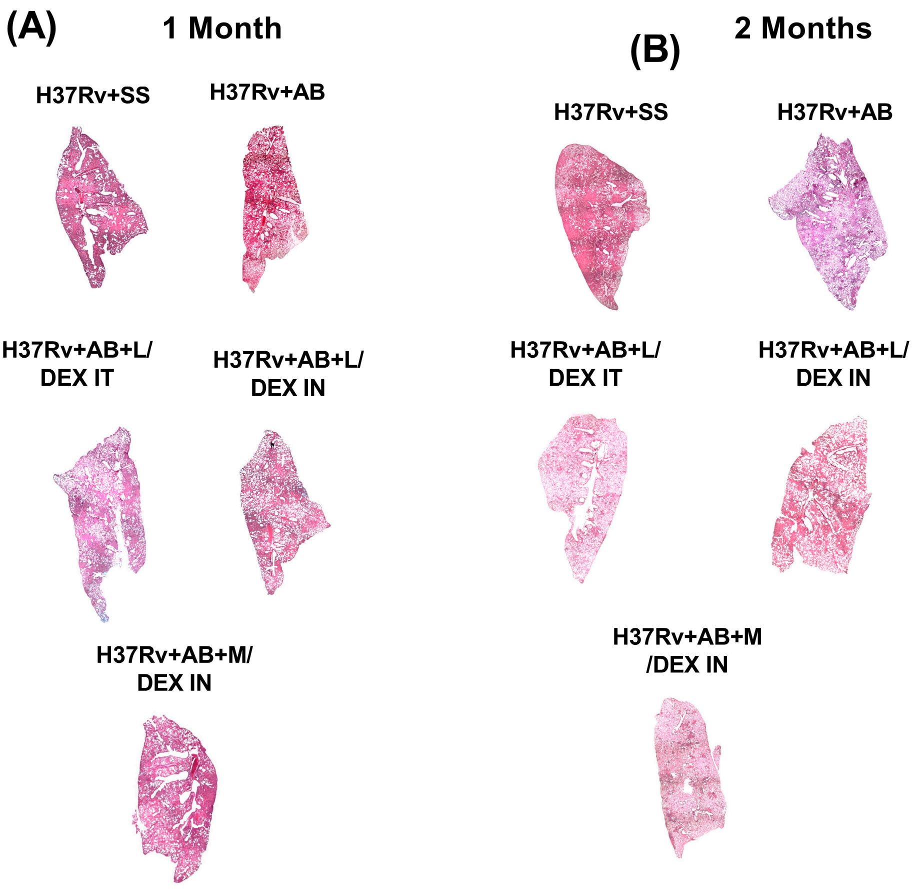

Figure S1. Effect of L/DEX and M/DEX doses combined with AB in the lung area affected by pneumonia in the progressive TB model. Representative micrographs of whole lungs after one (A) and two months (B) of DEX administration.

Table S1. Neurological Severity Score (NSS) for mice.

Task	Description	Points	
		Success	Failure
Hypomobility	Lower spontaneous mobility of the experimental mouse as compared with that of the external control when placed (30 s) on the top of the table. Scored 1,2 or 3 regarding locomotion, and speed and vigors of movements.	0	1,2,3
Lateralized posture	Abnormal body position characterized by a persistent tendency to recline sideward	0	1
Flattened posture	Abnormal body position characterized by slow movements and dragging the body along the tabletop	0	1
Hunched back	The persistent presence of a crouched posture	0	1
Piloerection	Persistent rise of the back hair.	0	1

Ataxic gait	The tendency to sway, rock, or lunch to the side as the animal proceeds forward.	0	1
Circling	Spontaneous or forced (gently pushing with one finger) walking consistently to one side.	0	1
Tremors	Presence of fine, repetitive, oscillatory movements observed during movement.	0	1
Twitches	Abrupt body jerks.	0	1
Convulsions	Repetitive twitches followed by extensions of the hind limbs.	0	1
Respiratory distress	Presence of increased, irregular, respiratory movements accompanied by breathing sounds.	0	1
Passivity	Characterized by the decreased behavioural response (struggle and escape) when the animals are covered with the hand to restrain movement.	0	1
Hyperreactivity	Characterized by the exaggerated behavioural response (struggle and escape) when the animals are covered with a hand to restrain movement.	0	1
Irritability	Characterized by aggressive posture and bitting behaviour exhibited by the animals when covered with a hand to restrain movement.	0	1
Ptosis	Closure of dropping of the upper eyes.	0	1
Urination	An excessive amount of urine on the animal's body and the tabletop	0	1
Decreased body tone	Characterized by relatively less resistance to compression or flaccidity of the abdominal muscles, determined by gentle compression of the sides of the animal between the lower thorax and pelvis using the thumb and index finger.	0	1
Forelimb flexion	Failure to extend one forepaw fully when the animal is held by the tail to a height of 10 cm and slowly lowered to observe symmetry in the outstretching of both forelimbs while the mouse reached the wire-mesh.	0	1
Decreased muscle strength	Characterized by decrease resistance when the animal is placed off the grid and gently drawn backwards by the tail.	0	1
Body rotation	Rolling along the axis of its body when the animal is held by the tail.	0	1
Motor incoordination	Characterized by decrease capacity to move and remain for at least 10 sec on the inclined plane (45°) or to grasp the cord (30 cm above the tabletop) with limbs and tail, and to remain there for a least 10 sec.	0	1,2,3
Absence of equilibrium	Characterized by a decreased capacity to remain for at least 10 sec on a horizontal bar (1 cm wide). Four paws have to be on the bar.	0	1,2,3
Hypoalgesia	Lack of behavioural response when an arterial claw is placed at 1 cm from the base of the animal's tail.	0	1

Hyperalgesia	Exaggerated behavioural response (disproportional vocalization and bitting) when an arterial claw is placed at 1 cm from the base of the animal's tail.	0	1
Sensitivity in the whiskers	Behavioural response when animal's moustaches are touched with a hyssop	0	1
Sensitivity in the abdomen	Behavioural response when the animal in gently touched in the abdomen.	0	1
Olfactory sensitivity	Behavioural response when hyssop with alcohol is placed in the noise of the animal	0	1
		**Global Score**	**31**
